# HOTAIR‐EZH2 inhibitor AC1Q3QWB upregulates CWF19L1 and enhances cell cycle inhibition of CDK4/6 inhibitor palbociclib in glioma

**DOI:** 10.1002/ctm2.21

**Published:** 2020-04-29

**Authors:** Jin Shi, Shigang Lv, Miaojing Wu, Xianggan Wang, Yan Deng, Yansheng Li, Kuanxun Li, Hongyu Zhao, Xingen Zhu, Minhua Ye

**Affiliations:** ^1^ Department of Neurosurgery The Second Affiliated Hospital of Nanchang University Jiangxi P.R. China; ^2^ Department of Neurology The Second Affiliated Hospital of Nanchang University Jiangxi P.R. China; ^3^ Department of Neurosurgery Laboratory of Neuro‐Oncology Key Laboratory of Post‐trauma Neuro‐repair and Regeneration in Central Nervous System Ministry of Education Tianjin Key Laboratory of Injuries Tianjin Medical University General Hospital Tianjin Neurological Institute Variations and Regeneration of Nervous System Tianjin P.R. China; ^4^ Department of Medicine Medical College of Nanchang University Jiangxi P.R. China; ^5^ Department of Neurosurgery Tongji Hospital Huazhong University of Science and Technology Wuhan P.R. China

**Keywords:** β‐catenin, AQB, CDK4, CDK6, cell cycle, CWF19L1, palbociclib

## Abstract

**Background:**

Glioblastoma (GBM) is the most common primary tumor in the brain, and the median survival time for GBM patients is only about 14 months; therefore, there is an urgent need for new and more effective strategies. Since cell cycle disorder is a key factor in tumor progression and immortalization, there is great potential for controlling cell cycle disorders in tumor cells in GBM patients. We began to study a novel combination of AQB and palbociclib to evaluate its potential as a new therapeutic target.

**Methods:**

Protein mass spectrometry was used to identify the tumor suppressor genes up‐regulated by AQB.The effects of HOTAIR ‐ EZH2 inhibitor AQB and CDK4/6 inhibitor Palbociclib on glioma cells lines were examined in vitro and in vivo experiments.

**Results:**

The combination of AQB and palbociclib inhibitors has a more pronounced suppression effect on the cell cycle, especially gliomas with high expression of HOTAIR and EZH2 and low expression of CWF19L1. We performed protein mass spectrometry to identify AQB upregulated tumor suppressor genes and confirmed that CWF19L1 is regulated by H3K27ac through chromatin immunoprecipitation‐quantitative PCR results. Univariate and multivariate Cox regression analysis and database analysis were performed to suggest CWF19L1 is a good prognostic factor. Our experimental results suggested that CWF19L1 can be significantly upregulated by AQB and lead to degradation of CDK4/6, resulting in G1 arrest. The combination of AQB and CDK4/6 inhibitor palbociclib is more effective in inhibiting the growth of glioma than in the single drug, both in vivo and in vitro. Similarly, we found that both AQB and palbociclib can inhibit Wnt/β‐catenin signaling, and the combined use of the two inhibitors has a stronger inhibitory effect on tumor metastasis.

**Conclusions:**

The combination of AQB and CDK4/6 inhibitor palbociclib has been found to have significant antitumor effects, which is likely to become a new strategy for glioma treatment.

**Abbreviations**
AQB: AC1Q3QWB
BBB: blood‐brain barrier
CCK‐8: Cell Counting Kit‐8
CGGA: Chinese Glioma Genome Atlas
ChIP‐qPCR: chromatin immunoprecipitation‐quantitative PCR 
GBM: glioblastoma
H&E: hematoxylin and eosin staining
PDX: patient‐derived xenografts
p‐RB: phosphorylated retinoblastoma
RB: retinoblastoma
siRNA: small interfering RNA
TCGA: The Cancer Genome Atlas

## 1 BACKGROUND

Glioblastoma (GBM) is the most common primary tumor in the brain, and is the most proliferative and reversible primary human cancer.^1^ Although standard treatment strategies, including surgery, chemotherapy, and radiation therapy, have been widely used, the median survival time for GBM patients is merely approximately 14 months.^2^ Therefore, new and more effective strategies are urgently needed. In recent years, the gradual deepening of molecular characterization of glioma has provided an important basis for the study of specific targeted drugs.^3^

At the same time, we learned that genes such as P53, P21, P16, and PTEN are common tumor suppressor genes in gliomas and are closely related to the cell cycle. Sinomenine promotes p53 expression and acetylation, leading to G0/G1 cell cycle arrest and apoptosis.^4^ P21 acts as a tumor suppressor gene to stop the cell cycle and limit cell proliferation.^5^ Furthermore, 40‐50% of gliomas have PTEN inactivation, leading to the abnormal activation of PI3K activity and downstream signaling pathways.^6^ In vitro and in vivo, PTEN also inhibits glioma cell growth.^7^ P16 causes cell cycle arrest at the G1‐S transition point.^8^ These above tumor suppressor genes are correlated to the cell cycle. It is also known that the abnormal regulation of the cell cycle leads to proliferation and genomic instability, and affects the occurrence and development of human cancer. AC1Q3QWB is a selective and efficient disruptor of the HOTAIR‐EZH2 interaction, which blocks the PRC2 recruitment and increases tumor suppressor expression.^9^ Through the series of tumor suppressor genes described above, the role of tumor suppressor genes in the cycle emphasizes the importance of targeted therapy in GBM. In fact, the cell cycle process is affected by many cyclin‐dependent kinases (CDKs), of which CDK4 and CDK6 play a key role in regulating cell proliferation by regulating the process of cells entering the DNA synthesis phase of the cell cycle.^10^ Since AQB can cause the expression of CWF19L1 to rise significantly, so we doubt whether CWF19L1 also has a related cycle inhibitory effect.

CDK4/CDK6 inhibitor palbociclib reduces tumor growth by reducing retinoblastoma (RB) protein phosphorylation and cell cycle arrest, which induces G1/S phase transition.^11^ In recent years, CDK4/CDK6 inhibitors have become a powerful drug for the treatment of cancer.^12^ However, a single application of palbociclib may not achieve the desired therapeutic effect, such as resistance. This means that the choice of combination may be more appropriate, and the effectiveness of the combination of palbociclib and other inhibitors has been demonstrated in various models. For example, CDK4/CDK6 inhibitors, combined with endocrine therapy, have good clinical activity against metastatic estrogen receptor‐positive.^13^ It was found that CDK4/CDK6 inhibitors can be combined with a variety of drug treatment models that have been investigated in preclinical models of various tumor types, and many of which are presently undergoing clinical trials (http://www.clinicaltrials.gov), which include palbociclib and letrozole in HR+/HER2‐negative operable breast cancer, palbociclib in combination with tamoxifen as the first line therapy for metastatic hormone receptor positive breast cancer, palbociclib with fulvestrant for metastatic breast cancer, and palbociclib and cetuximab in metastatic colorectal cancer. Significant advances have also been made in the use of related pathway inhibitors, such as the antiproliferative effect of CDK4/CDK6, and the mTOR inhibitor temsirolimus in diffuse pons glioma cells in vitro.^14^ The binding of CDK4/CDK6 to the mTOR inhibitor everolimus exhibits a synergistic effect on anti‐GIC and induces apoptosis in vitro.^15^ In aggressive and therapeutically resistant thyroid cancer, PI3K/mTOR and CDK4/CDK6 inhibitors have a significant effect.^16^ Given the feasibility of a combination therapy regimen in different tumors, we believe that the combined use of AQB and palbociclib is also likely to induce an important antitumor effect.

In the present study, the in vitro experiments revealed the efficacy and mechanism of action AQB in combination with palbociclib on cell cycle inhibition in glioma cell lines, with a high expression of HOTAIR and EZH2, and a low expression of CWF19L1. In the GBM patient‐derived xenograft (PDX) models, AQB and palbociclib were observed to have potent antitumor effects, and combined treatment is more effective than treatment alone. These results provide evidence to support the clinical trials of these combination therapies in glioma patients.

## 2 RESULTS

### 2.1 AQB promotes the expression of tumor suppressor gene CWF19L1

**FIGURE 1:**
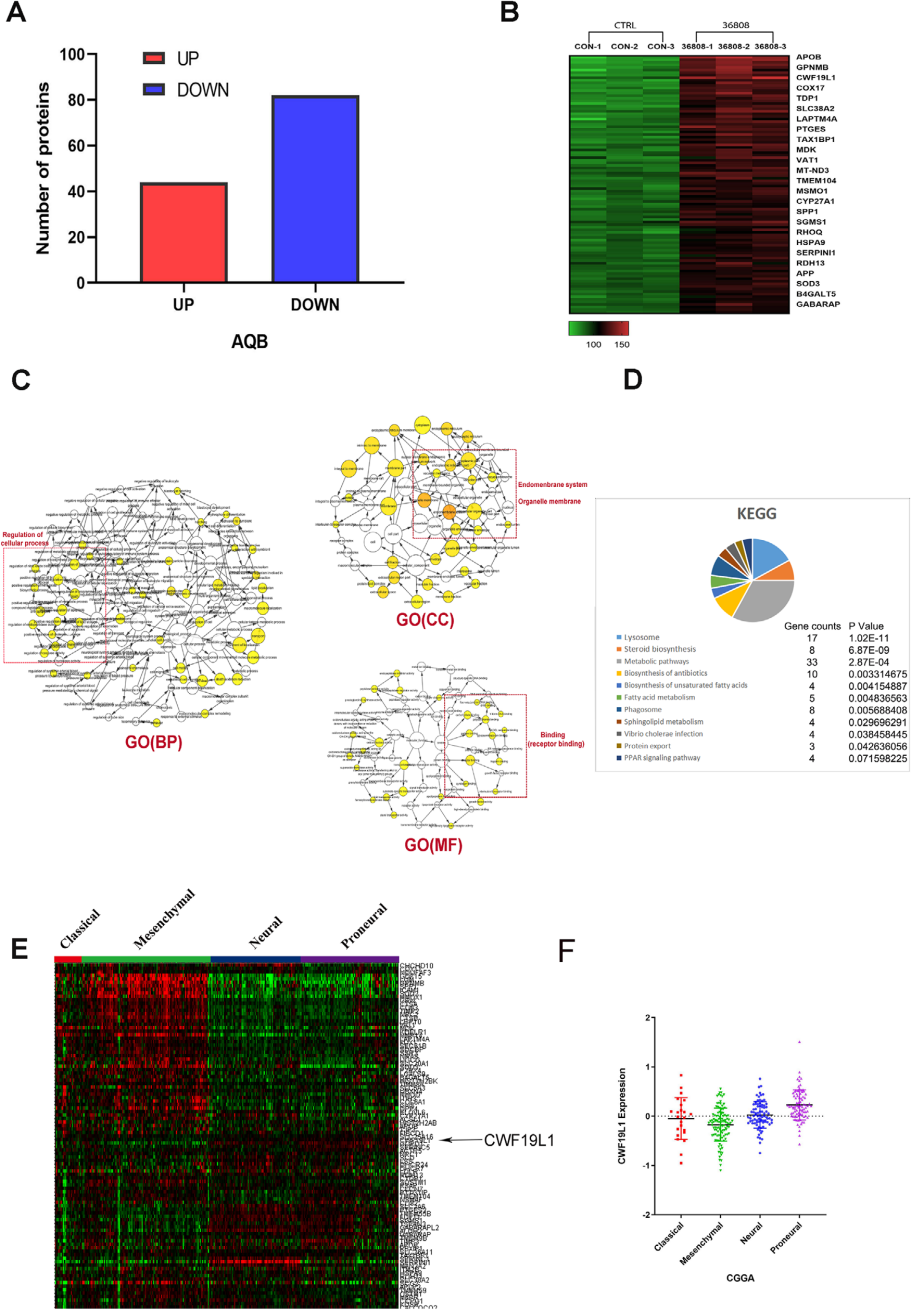
AQB can promote the expression of tumor suppressor genes, and CWF19L1 is a gene of research value. A and B, After AQB treatment, the protein profiling revealed a statistical difference of more than 1.5 protein number and expression. C, The analysis of UP histone function through the Cytoscape software was associated with the proliferation and apoptosis. D, The TCGA database cluster analysis (selecting the top 100 genes in the UP group) E, The analysis of CWF19L1 levels from the TCGA data obtained from different glioma subtypes revealed that CWF19L1 was more highly enriched in patients with anterior subtypes

Since AQB is a selective and effective disruptor of the HOTAIR‐EZH2 interaction, it was hypothesized that this inhibitor could promote the expression of related tumor suppressor genes. We initially explored protein profiling performed 2 days after AQB treatment. Totally, 44 genes were upregulated in U87cells with adjusted *P* value <.05 and fold change larger than 1.5 (Figure 1A). The gene ontology (GO) analysis revealed that these genes are associated with proliferation and death (Figure 1C). The Kyoto Encyclopedia of Genes and Genomes (KEGG) analysis revealed that CWF19L1 is involved in functions related to cellular processes. To gain insight into profile of CWF19L1 in glioma samples, we employed Chinese glioma genome atlas (CGGA) database data (100 selected genes) for cluster analysis and found that CWF19L1 had higher levels of expression in proneural glioma than the other three GBM subtypes (classic, mesenchymal, nerve), because the proneural glioma has a good prognosis.^17^ We suggest that CWF19L1 may be associated with a better prognosis of glioma (Figure 1E and F, *P* < .0006). In the UP group, CWF19L1 ranked 10th in research value (Figure 1B).

### 2.2 CWF19L1 expression is associated with the grade of glioma and the prognosis of patients

**FIGURE 2:**
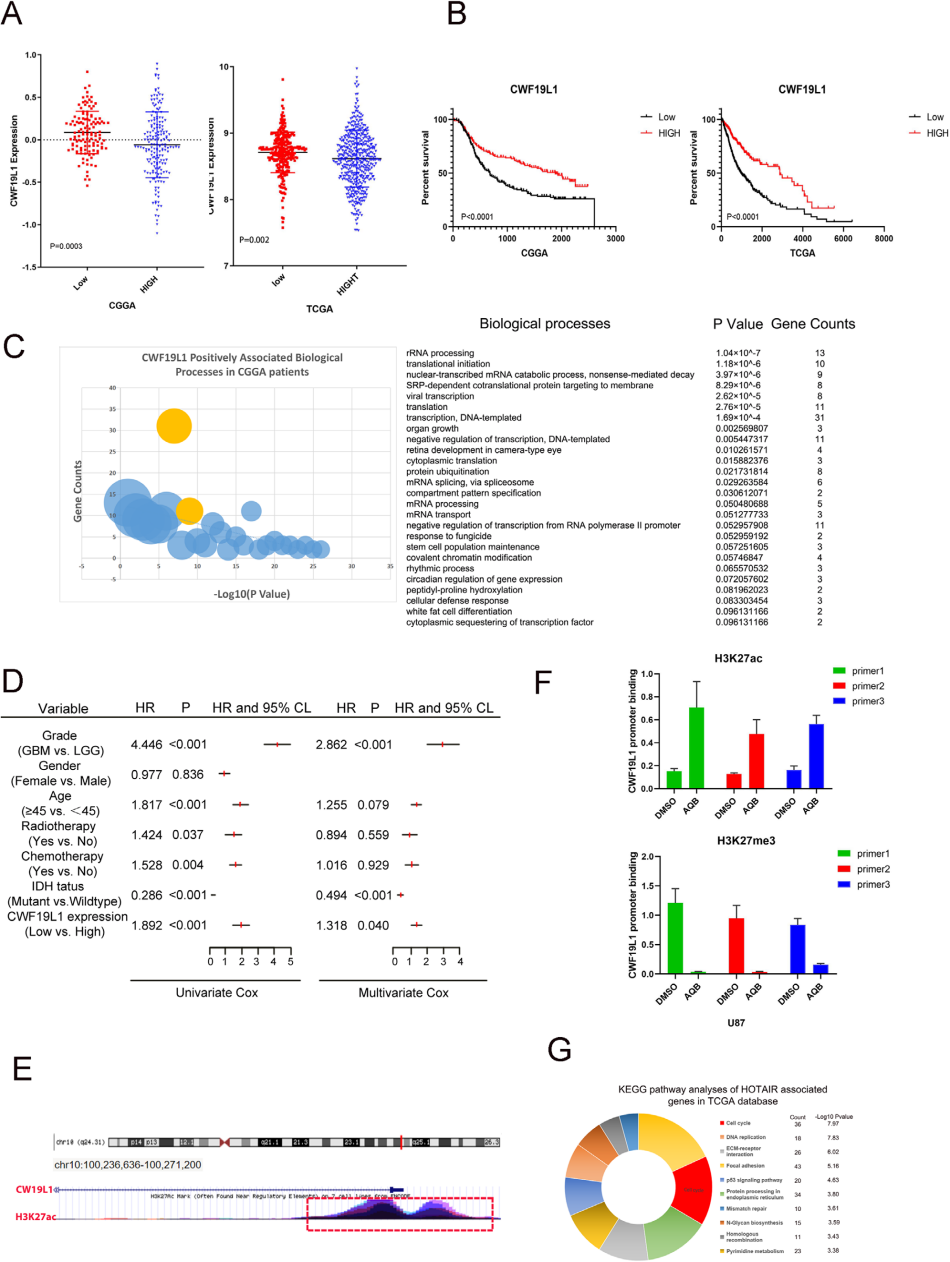
The expression of CWF19L1 is associated with the grade of glioma and confers a better prognosis for patients with glioma. A, The expression levels of CWF19L1 in the TCGA and CGGA RNAseq data sets were negatively associated with the glioma World Health Organization (WHO) grades. B, The Kaplan‐Meier curve shows the high expression of CWF19L1, suggesting that patients have a better prognosis. C, The positive correlation of CWF19L1 in CGGA patients suggests a higher correlation with the transcriptional process. D, The Cox proportional hazards regression analysis of CWF19L1 expression and other characteristics in relation to overall survival in GBM from the CGGA cohort. E, The UCSC website predicts that the CWF19L1 promoter region is rich in H3K27ac. F, ChIP‐qPCR showed the enrichment of H3K27ac in the CWF19L1 promoter region. Interestingly, H3K27me3 showed the opposite phenomenon. H, The GO analysis of HOTAIR positive correlation genes through the TCGA database

Gliomas were classified as grade II, III, or IV, according to the World Health Organization (WHO) classification criteria. RNAseq databases from The Cancer Genome Atlas (TCGA) and CGGA cohorts were used to reveal the CWF19L1 and glioma grade correlation. As shown in Figure 2A, the expression level of CWF19L1 was significantly correlated with the tumor grade (TCGA *P* < .0006 and CGGA *P* < .002). Subsequently, the prognostic value of CWF19L1 in TCGA and CGGA was further evaluated using the Kaplan‐Meier survival curve analysis with log‐rank comparison. The analysis results revealed that patients with high expression of CWF19L1 have higher survival rates in the TCGA and CGGA databases (Figure 2B, *P* < .0001), By immunohistochemical analysis of glioma normal brain tissues (NBTs), low‐grade glioma, and high‐grade glioma, we found that the expression level of CWF19L1 is highest in glioma paracancerous tissue but lowest in high‐grade glioma (Figure S1).

Next, we found that high CWF19L1 expression, older age, IDH1 mutation, radiotherapy, and chemotherapy were associated with overall survival by performed univariate Cox regression analysis of GBM patients from the CGGA cohort (Figure 2D). The further analysis by the multivariate Cox proportional hazards model suggested that CWF19L1 expression was independently associated with overall survival (Figure 2D, hazard ratio [HR] = 1.318, *P* < .040). In conclusion, the expression of CWF19L1 is negatively correlated with the WHO grading of glioma, while the high expression of CWF19L1 is associated with a better survival prognosis. These analyses indicate that CWF19L1 is a promising independent biomarker for the diagnosis of glioma. In addition, in order to understand why the expression of CWF19L1 is increased after AQB (40µM) treatment, we collected information of the regulation of histone modification in the CWF19L1 promoter region from the UCSC genome browser (Figure 2E). In the CWF19L1 promoter region, we found that it is rich in many H3K27ac modifications. As we all know, H3K27ac and H3K27me3 modification are based on the same lysine residue, so some studies have found that H3K27me3 antagonizes the multicomb‐inhibiting complex 2‐dependent gene silencing caused by acetylation of H3K27 (H3K27ac). In addition, acetylation and trimethylation of H3K27 are in a competitive relationship.^17^, ^18^ As our previous research has shown that AQB can reduce the trimethylation of H3K27, we believe that AQB can indirectly increase the acetylation of H3K27 by reducing the trimethylation of H3K27, thereby inhibiting the silencing of the relevant tumor suppressor gene. In order to assess the chromatin status of the CWF19L1 promoter in the GBM cell line, chromatin immunoprecipitation‐quantitative PCR (ChIP‐qPCR) analysis was performed using antibodies against H3K27ac and four genomic PCR primers against the CWF19L1 promoter. Compared to the DMSO control group, the number of H3K27ac in the binding promoter region of the AQB treatment group increased after treatment with AQB (40 µM). In contrast to H3K27ac, H3K27me3 has less binding in the promoter region of the AQB treatment group (Figure 2F).

These results confirmed that the transcription of CWF19L1 is regulated by H3K27ac histone modification. In order to understand the related functions of CWF19L1, the CGGA RNAseq database was used to search for the top 2000 genes that positively correlated with CWF19L1. Then, a GO analysis was performed on the database for annotation, visualization and integrated discovery (DAVID) software, and the biological process ontology was classified. It was found that CWF19L1 and the transcriptional biology processes are highly correlated (Figure 2C). It is known that many oncogene transcriptional activation and transcription factor–mediated pathway activations play a very important role in the occurrence and progression of cancer. Hence, we speculated that CWF19L1 also affects tumor formation and proliferation. In the subsequent data analysis, we also founded that the GO analysis of the HOTAIR positive correlation gene in the TCGA database is closely correlated to the cell cycle (Figure 2G), and the literature indicates that HOTAIR expression and gene set–associated cells are involved in the cell cycle progression.^19^ Hence, we believe that CWF19L1 is likely to be involved in cell cycle regulation.

### 2.3 The key role of CWF19L1 in the proliferation of glioma cells

**FIGURE 3:**
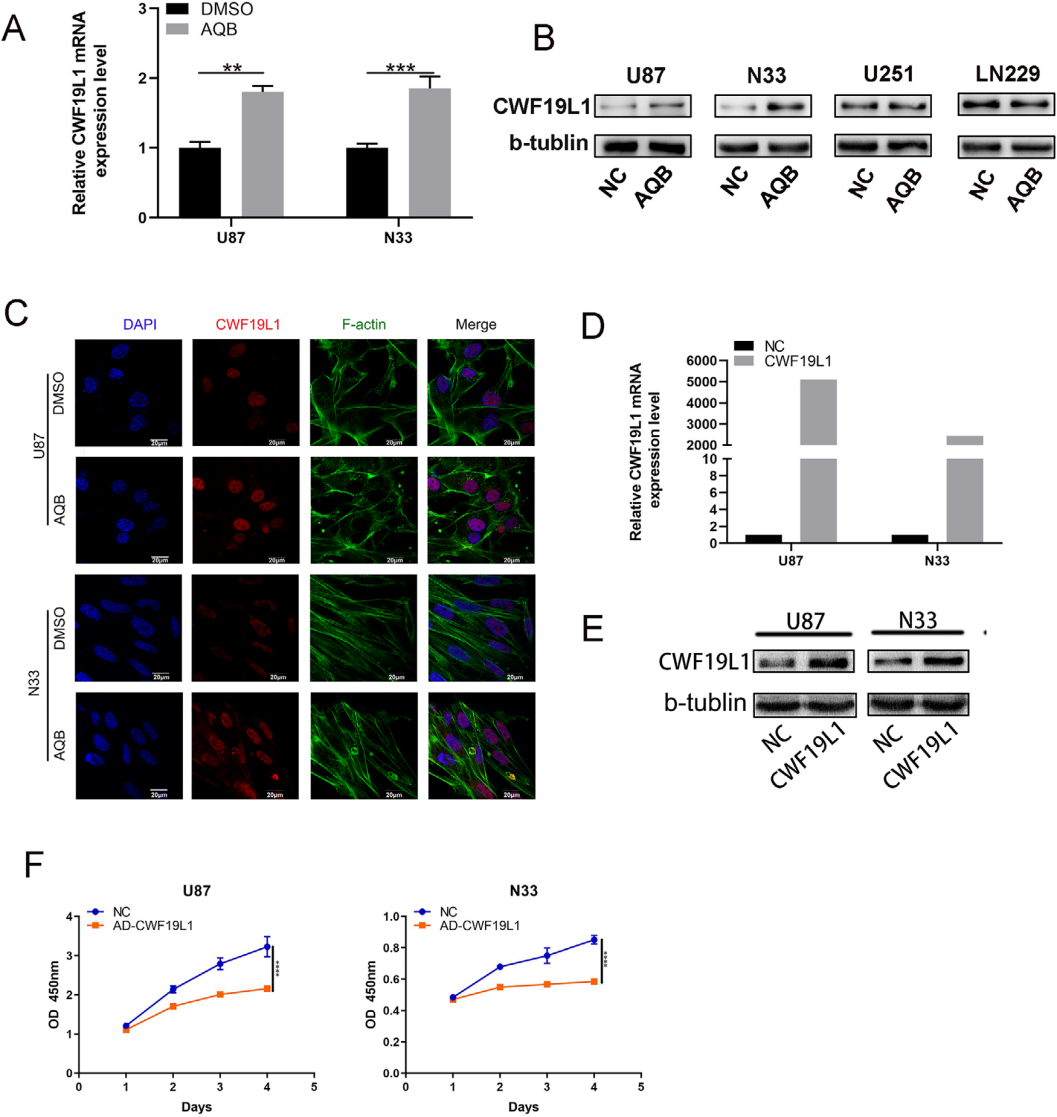
The key role of CWF19L1 in the proliferation of glioma cells. A, The western blot data revealed the upregulation of CWF19L1 protein levels in U87 and N33 cells, while CWF19L1 expression levels were not significantly different in U251 and LN229. B, The upregulation of CWF19L1 mRNA levels in U87 and N33 cells after AQB treatment (^*^ *P* < .05, ^**^ *P* < .01, ^***^ *P* < .001). C, The confocal microscopy revealed that AQB upregulated CWF19L1 and remodeled the actin (F‐actin) cytoskeleton. DAPI was used to stain the nuclei. Bar, 20 um. E, CWF19L1 expression increased in the overexpression group. F, Detection of the proliferation of U87 cells by CCK‐8 assay after the transfection of the CWF19L1 plasmid

The data analysis revealed that CWF19L1 is associated with a better prognosis in patients with glioma. This prompted us to investigate its biological role in glioma. In order to further determine whether AQB could result in an increased expression of CWF19L1, after treatment with AQB (40 µM) and DMSO for 2 days in the U87 and N33 cell lines, RT‐qPCR showed that the expression of CWF19L1 in the AQB group was significantly increased compared with the negative control DMSO group (Figure 3A). Furthermore, the western blot data revealed the upregulation of CWF19L1 protein levels in U87 and N33 cells, while CWF19L1 expression levels were not significantly different in U251 and LN229 (Figure 3B), so we selected U87 and N33 cell lines for in vitro experiments. In addition, the confocal microscopy revealed the increased expression of CWF19L1 in both cells (Figure 3C). Therefore, it can be confirmed that AQB promotes the expression of CWF19L1. Next, a special plasmid against CWF19L1 was transfected into U87 and N33 cells to change the expression level of CWF19L1, and the success of the transfection was confirmed by immunofluorescence and RT‐qPCR (Figure 3 and E). The Cell Counting Kit‐8 (CCK‐8) assay was used to assess the short‐term cell viability. Compared with the control group, the transfection group strongly inhibited the cell viability (Figure 3F).

### 2.4 The key role of CWF19L1 in the glioma cell cycle

**FIGURE 4:**
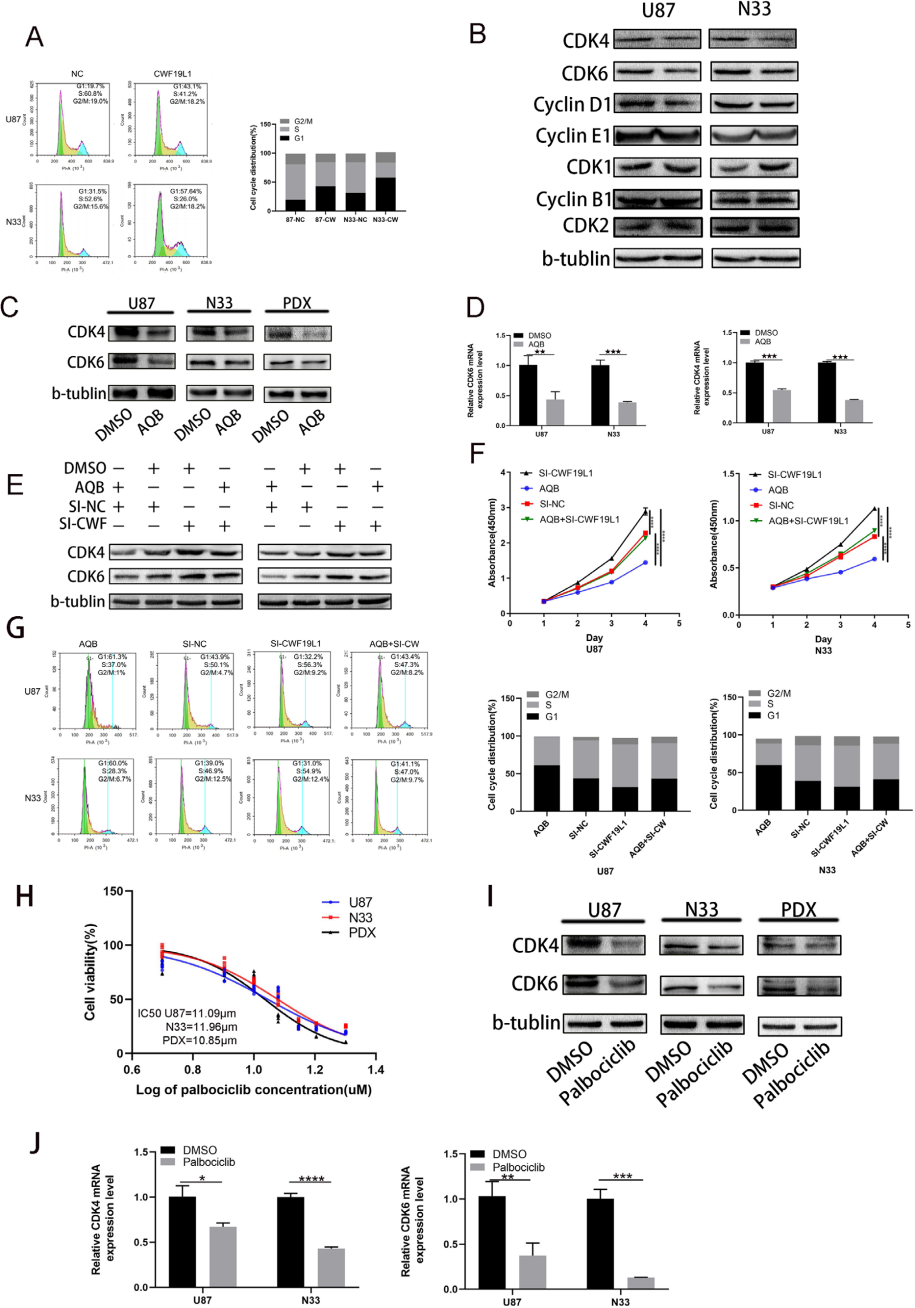
The significant role of CWF19L1 in the cell cycle of glioma. A, The flow cytometry analysis of the cell cycle stages of U87 and N33 cells transfected with the CWF19L1 plasmid and NC group plasmid. B, After 48 hours of U87 and N33 cell overexpression of the CWF19L1 plasmid, the western blot of CDK1, CDK2, cyclinE1, and cyclinB1 did not significantly change, when compared with the NC group. The CDK4, CDK6, and cyclinD1 expression decreased, with β‐actin as a loading control. C and D, The qPCR and western blot data of CDK4 and CDK6 expression in glioma cells after treatment with AQB. E and F, The rescue experiment by adding SI‐CWF19L1 in the presence or absence of AQB in glioma cells, the western blot analysis of CDK4 and CDK6 expression in U87 and N33 cells, the cell viability of U87 and N33 cells transfected with SI‐CWF19L1, and the use of AQB separately or combined were detected using by CCK‐8 assay. G, The flow cycle analysis of the cell cycle distribution of glioma cells. H, The IC50 of CDK4/CDK6 inhibitor palbociclib. I and J, The qPCR and western blot data revealed the CDK4 and CDK6 expression in glioma cells at 2 days after AQB treatment (^*^ *P* < .05, ^**^ *P* < .01, ^***^ *P* < .001, ^****^ *P* < .0001)

Since previous analysis showed that there may be a link between CWF19L1 and the cell cycle, and the decline in cell proliferation is usually accompanied by changes in cell cycle progression, we subsequently investigated cell cycle–related changes. The flow cytometry analysis revealed that the overexpression of CWF19L1 inhibited the G1/S conversion (Figure 4A). In addition, western blotting showed that cell cycle–associated CDK4 and CDK6 protein expression levels were reduced by transfection with the CWF19L1 plasmid, when compared to the control group (Figure 4B). Furthermore, western blot and RT‐qPCR suggested the significant decrease in CDK4 and CDK6 expression (Figure 4C and D). Since CWF19L1 has been shown to be regulated by H3K27ac, this indicates that AQB causes an increase in CWF19L1. Then, we transfected SI‐CWF19L1 into U87 and N33 cells, and treated with AQB (40 µM) and DMSO for 2 days, as shown in Figure 4E. This exhibited a decrease in CDK4 and CDK6 level due to the AQB treatment, and this was saved by the knockdown of CWF19L1. In order to further confirm whether CWF19L1 is an important target for AQB in the cell cycle, we performed a recovery‐related cell proliferation experiment. As shown in Figure 4F, the AQB inhibition of cell cycle status was reversed by si‐CWF19L1. These results indicate that CWF19L1 is a cycle‐related functional target of AQB in GBM cells. Subsequently, we analyzed the effect of palbociclib, another cell cycle inhibitor, on the cell cycle inhibition of the G1 to S phase. The IC50 of the initial U87 and N33 cell lines was 11 µM and 12 µM, respectively (Figure 4H); as shown in Figure 4I and J, the U87 and N33 cell lines were treated with palbociclib (11 µM and 12 µM) and DMSO for 2 days; and the immunoblotting and RT‐qPCR revealed that CDK4 and CDK6 expression was reduced, when compared to the negative control DMSO group.

### 2.5 The combined application of AQB can enhance the inhibition of the proliferation of palbociclib in vitro

**FIGURE 5:**
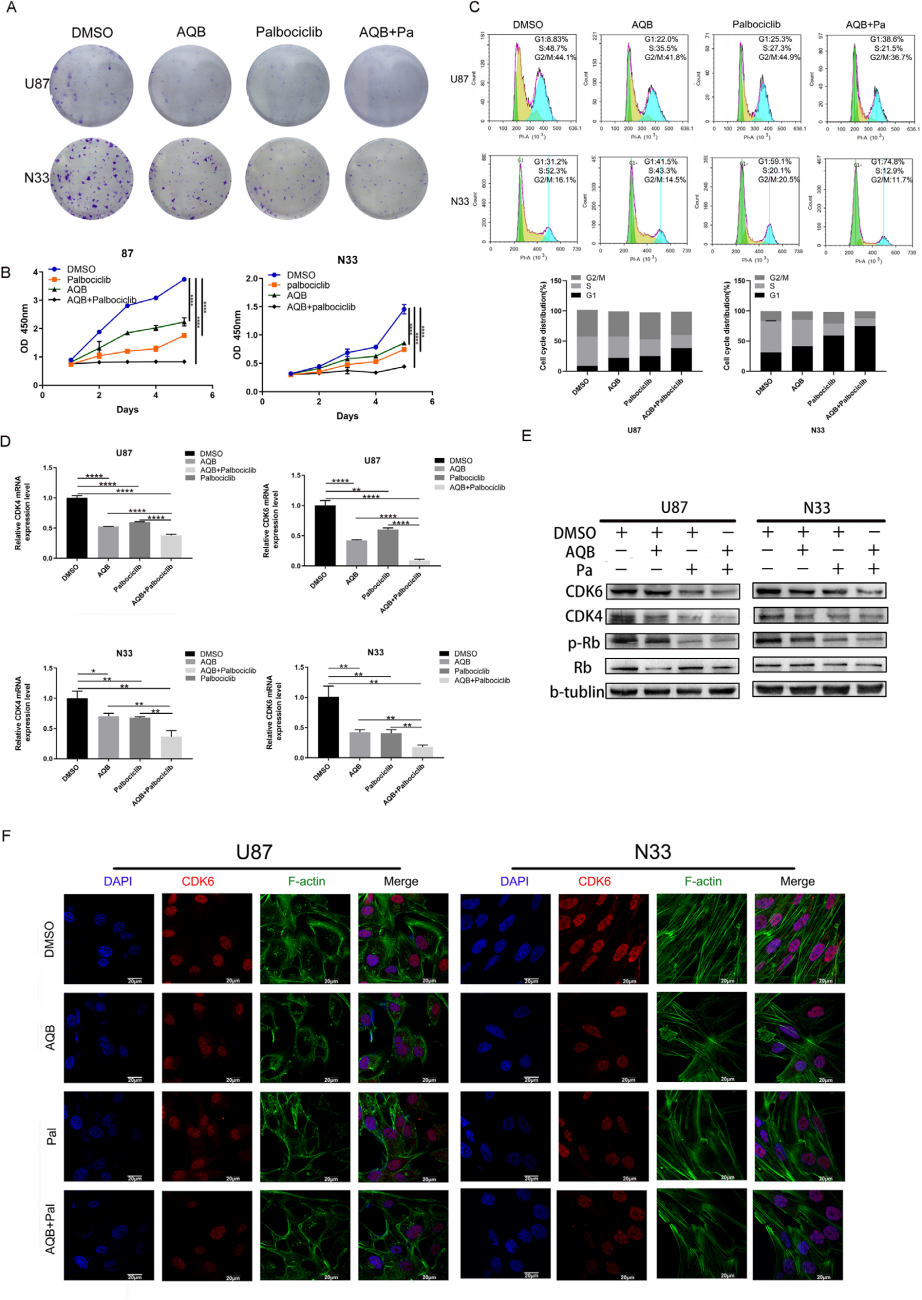
Effect of the combination of palbociclib and AQB inhibitors on the cell cycle. A and B, The colony formation assays and CCK‐8 experiments demonstrate the effect of DMSO, AQB, palbociclib, and combination therapy on the cell proliferation in glioma cells. C, The cell cycle distribution of U87 and N33 was measured using flow cytometry. D and E, The qPCR and western blot data confirmed that the decrease of CDK4, CDK6, and p‐RB in glioma cells was more pronounced after the combined administration (^*^ *P* < .05, ^**^ *P* < .01, ^***^ *P* < .001, ^****^ *P* < .0001). F, The confocal microscopy images of F‐actin (filamentous pseudopodia formation of F‐actin) and CDK6 in N33 and U87 cells using AQB and palbociclib alone, or in combination. DAPI was used to stain the core. Bar, 20 mm

Initially, the IC50 of palbociclib in the U87 and N33 cell lines by cell viability was determined. We then assessed whether HOTAIR‐EZH2 inhibitor AC1Q3QWB had synergistic effect with CDK4/6 inhibitor palbociclib treatment. Compared with AQB or palbociclib alone, AQB plus Pa exhibited enhanced cytotoxicity in GBM cells. The confidence interval (CI) values were all <0.8, indicating a strongly synergistic interaction between AQB and palbociclib in GBM cells (Figure S2). Plate cloning experiments showed that that the combination of AQB and Pa showed better glioma cell inhibition of proliferation than the two inhibitors of AQB and Pa alone (Figure 5A). Similarly, the CCK‐8 results revealed that for both glioma cells, the combination drug revealed a more significant inhibitory effect on proliferation, when compared to the drug alone (Figure 5B). In order to understand how AQB and palbociclib inhibit the proliferation of U87 and N33 cells, we also performed cell cycle experiment, and the results revealed that in the DMSO, AQB, palbociclib, and combination treatment groups, the combination treatment group significantly inhibited the G1 to S phase transition, leading to G1 cell cycle arrest (Figure 5C). The results of apoptosis assay proved that the combination of two inhibitors has a more significant effect on the promotion of U87 and N33 cell apoptosis than when the inhibitors were used alone (Figure S3). The subsequent western blot revealed that AQB or palbociclib alone inhibited the expression of CDK4, CDK6, and phosphorylated RB, and the effect was more pronounced after the combined application of these two inhibitors (Figure 5C and D). Similarly, the confocal microscopy revealed that CDK6 was mainly expressed in the nucleus of both cells, and these observed results were consistent with the immunoblotting results (Figure 5E), which fully demonstrate that small molecule inhibitor AQB can enhance the inhibitory effect of CDK4/6 inhibitor palbociclib on the cell cycle. In particular, this can lead to G1 arrest in glioma cells, thereby inhibiting cell proliferation.

### 2.6 The combined application of inhibitors can enhance the inhibition of Wnt/β‐catenin signal transduction in vitro

**FIGURE 6:**
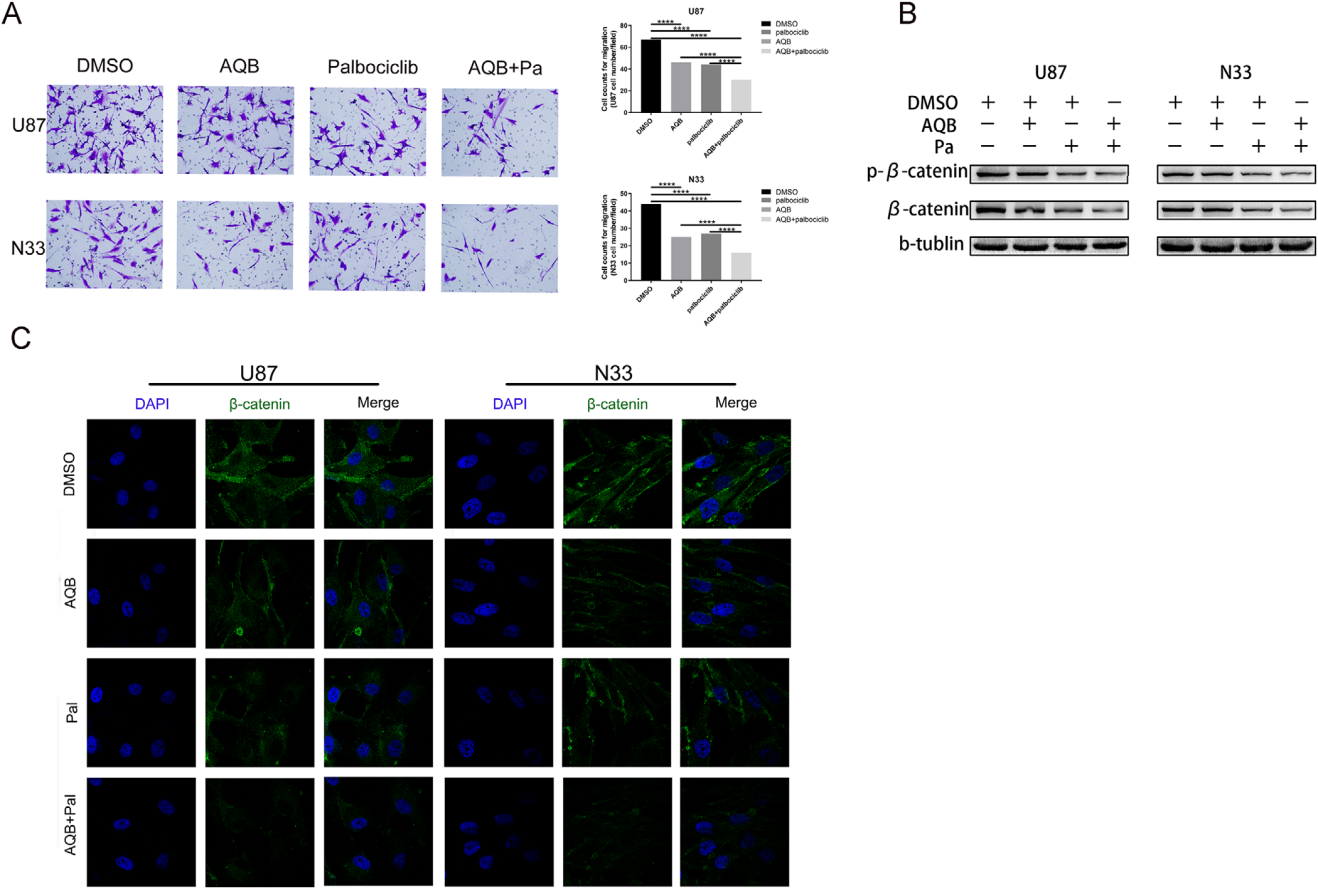
The combined application of AQB enhanced the inhibitory effect of palbociclib on wnt/β‐catenin signaling in vitro. A, The Transwell analysis demonstrated that cell invasion was inhibited by U87 and N33 cells alone or in combination (^*^ *P* < .05, ^**^ *P* < .01, ^***^ *P* < .001, ^****^ *P* < .0001). B, Immunoblot analysis of the effects of small molecule inhibitors on wnt/β‐catenin signaling in glioma cell lines, either alone or in combination. C, The confocal microscopy revealed the changes in the expression of β‐catenin after drug treatment in both cell lines

The effects on the cell cycle and proliferation have been confirmed in the above results. Hence, we tempted to investigate the effects of these two small molecule inhibitors on invasion and migration. Previous studies have revealed that AQB can cause the degradation of β‐catenin and inhibition of Wnt/β‐catenin signaling.^9^ It was also found that palbociclib reduces Ser9‐GSK3β phosphorylation, increases its stability, and ultimately induces β‐catenin degradation.^20^ Hence, the Transwell method was used to test migration inhibition effect of DMSO, AQB, palbociclib, and the combination treatment on cancer cells, and it was found that the combination treatment exhibited the most obvious inhibition, and this also directly indicates that the combined treatment would be at a more advantageous level (Figure 6A). Compared with the use of small molecule inhibitors alone, immunoblotting revealed that the combination of AQB and palbociclib significantly inhibited β‐catenin and p‐β‐catenin (Figure 6B). The confocal analysis revealed that the combination of AQB and palbociclib induced a lower level of β‐catenin protein, when compared to either drug alone (Figure 6C).

### 2.7 The combination of AQB and palbociclib inhibited tumor formation in vivo

**FIGURE 7:**
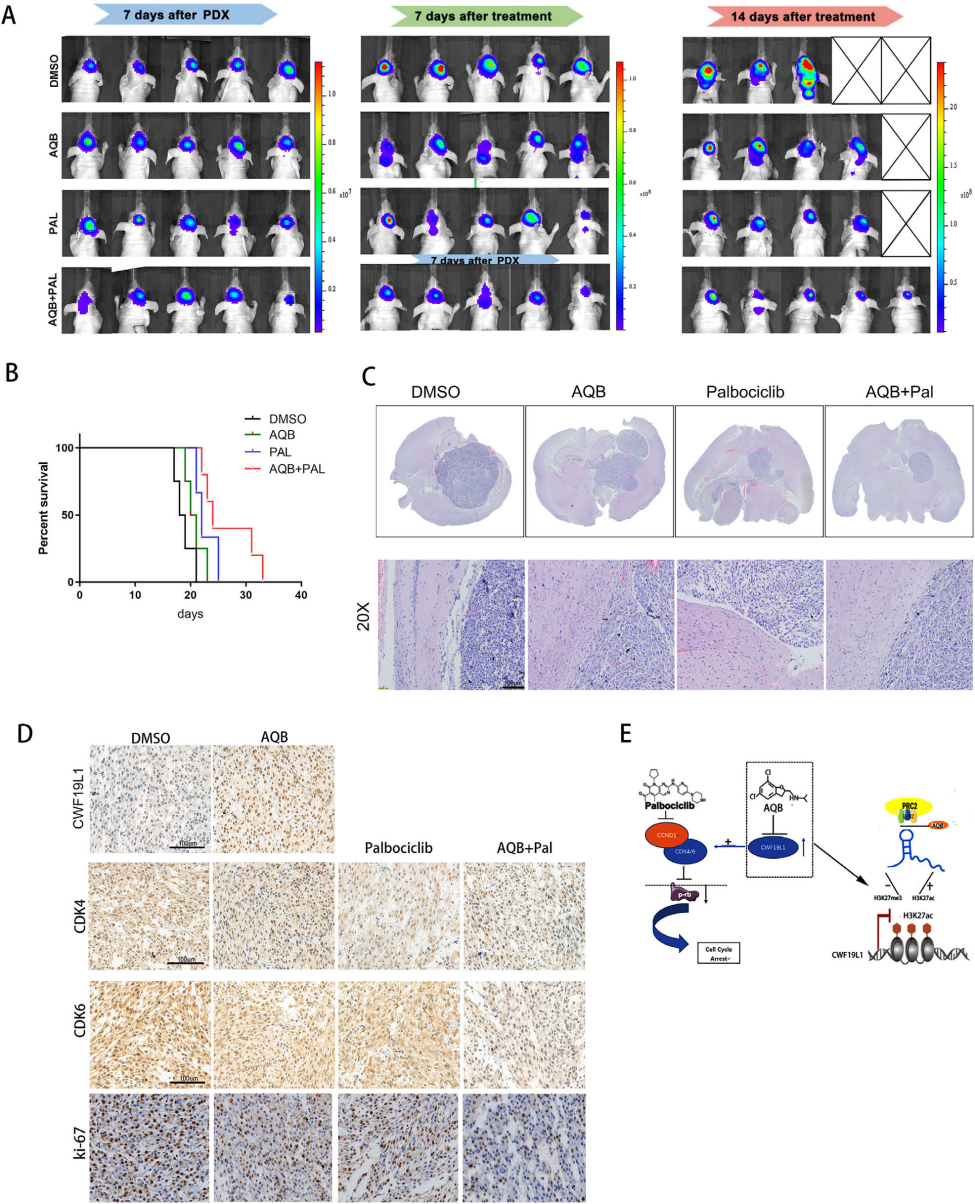
Potent antitumor efficacy of palbociclib and AQB in tumor‐bearing mice. A, The glioblastoma PDX mice were randomly divided into four groups, and each group was treated with DMSO, AQB (100 mg/kg), palbociclib (100 mg/kg), AQB (100 mg/kg), and palbociclib (100 mg/kg), respectively. Bioluminescence was performed once a week, and the bioluminescence revealed tumor growth. B, The Kaplan‐Meier survival curve revealed the overall survival of tumor‐bearing mice. C, The H&E‐stained images of intracranial tumor tissue sections obtained from nude mice from the different treatment groups. D, The tissue micrographs of the four groups, and the representative micrographs of the immunohistochemistry using antibodies against CWF19L1, CDK4, CDK6, and Ki67 are shown; scale bar, 100 µm. E, The mechanism suggested by the results of the study

In order to further understand the therapeutic effects of the AQB/palbociclib combination in vivo, we constructed a GBM patient–derived xenograft (PDX) model. After establishing the tumor model, mice were randomly divided into four groups and administered with DMSO, AQB (100 mg/kg), palbociclib (100 mg/kg), and AQB (100 mg/kg)/palbociclib (100 mg/kg) every 2 days. A single dose was given. The PDX cells used to establish the GBM model were derived from the patient's GBM tissue. The bioluminescence images taken after 7, 14, and 21 days after gavage treatment revealed that the tumors were much smaller in the combination treatment group, when compared to those who received the single drug alone (Figure 7A). Furthermore, it was apparent that mice in the combination treatment group had higher survival rates than any of the drug treatments alone (Figure 7B). The hematoxylin and eosin (H&E) staining analysis of the mouse brain tumor sections revealed that the tumor size was much smaller in the combined group than in the control group or mice treated with any one drug, and the tumor boundaries were more clear (Figure 7C). The immunohistochemistry results revealed that the expression of Ki67, CDK4, and CDK6 was lower in the combined treatment group than in the single‐agent and DMSO control groups.The AQB‐treated group had a significantly greater CWF19L1 expression, when compared to the DMSO group (Figure 7D). These data indicate that AQB does increase the expression of CWF19L1, and that the therapeutic combination of AQB and palbociclib can effectively inhibit tumor proliferation and metastasis, which also provides a new therapeutic opportunity for glioma in the combination therapy with AQB and palbociclib.

## 3 DISCUSSION

GBM is the most malignant histological type of glioma, and the prognosis is generally poor.^21^ Although certain research and clinical trials have been conducted in the past decade, its effective treatment remains limited. Hence, understanding the mechanisms associated with GBM tumor infiltration, proliferation, and metastasis has become very important. Epigenetic studies reveal tumorigenesis. Among them, crucial tumor suppressor gene silencing or oncogene expression upregulation is often one of the important factors leading to the development of cancer, so we believe that epigenetics will play a key role in the future diagnosis and treatment of cancer.^22^

In the present study, a protein profile analysis was conducted after 2 days of AQB treatment. We found that CWF19L1 is a cell cycle–related gene, and that it has a good antitumor effect when combined with palbociclib. Previous experiments have revealed that AQB can reverse the HOTAIR‐PRC2‐mediated epigenetic gene silencing by specifically disrupting and blocking the HOTAIR‐EZH2 interaction.^9^ To better understand the mechanism by which this mechanism may reverse the inhibition of the tumor suppressor gene, we performed protein profiling and found that CWF19L1 has important research value in upregulating proteins. A study only revealed the role of CWF19L1/hDrn 1 in an intron turnover after hDbr1 splicing,^23^ and a report corroborates that loss‐of‐function mutations in CWF19Ll lead to early onset cerebellar ataxia and (progressive) cerebellar atrophy.^24^ To better understand the functions of CWF19L1, we analyzed the GO expression and prognosis in TCGA and CGGA, and the GO analysis of CWF19L1 positively correlated genes. The biological process ontology labeling classification prompts and transcriptional biology processes were correlated. In fact, the biological process of transcription was closely correlated to the progression of tumors. Furthermore, common genetic variations in gene regulatory elements recognized by HOX transcription factors may lead to increased susceptibility to cancer.^25^ MYC stimulates the transcription of DANCR, and DANCR limits the expression of cell cycle inhibitor p21 (CDKN1A),^26^ thereby promoting the proliferation of cancer cells. Therefore, the transcriptional activation of many oncogenes and transcription factor–mediated pathway activations play a very important role in the occurrence and progression of cancer. The promoter region of CWF19L1 is regulated by H3K27ac through chip‐qPCR. This prompted us to further determine its related mechanism in glioma. Next, we found that overexpression of this gene can inhibit the proliferation of glioma cells, thereby confirming its inhibitory effect on the proliferation of glioma cells. Furthermore, cell cycle disorders can lead to uncontrolled cell proliferation and ultimately tumorigenesis.^27^, ^28^, ^29^ A previous literature on HOTAIR and cell cycle reported that the reduction in HOTAIR expression resulted in a significant increase in cells in the G0/G1, and that AQB is a potent blocker of HOTAIR‐EZH2. The chip‐qPCR technique also confirmed that the promoter region of CWF19L1 is regulated by H3K27ac. Based on the above results, we further determine that CWF19L1 is related to the cell cycle.

In order to understand the role of CWF19L1 in the cell cycle, from the results of flow cytometry and western blot, we can see that CWF19L1 can inhibit the G1‐to‐S phase transition. Since CDK activation and inactivation regulate the conserved regulatory mechanisms in eukaryotes from G1 into S phase, G2 enters M,^30^ we explored the changes of cell cycle–related indicators in western blot experiments and found that the expression level of cyclin D1, CDK4, and CDK6 exhibited a downward trend. Interestingly, cyclin B1, cyclin E1, CDK1, CDK2, and other indicators did not change. This is sufficient to demonstrate that CWF19L1 can inhibit the progression of the cell cycle by enhancing the blockade of G1.

For the role of the drug in the cycle, it was found that AQB mediated the CDK4/CDK6 reduction, providing a theoretical basis for the drug combined with CDK4/CDK6 specific cytostatics. Palbociclib is a specific cytokine inhibitor of CDK4/CDK6 that binds to the ATP‐binding pocket of CDK4/CDK6, blocks RB phosphorylation, and inhibits the G1‐to‐S phase transition, resulting to G1 arrest.^31^ Since palmitic acid kinase has been approved for the treatment of breast cancer, as well as in various types of cancers,^32^, ^33^, ^34^, ^35^ we considered that this has more research value. It was demonstrated that the combination therapy inhibited the proliferation of U87 and N33 cells by plate cloning and CCK‐8 experiments, and it was found that the expression levels of CDK4 and CDK6 were more markedly decreased in the combination group than in the single drug group. Similarly, the same result was observed for the phosphorylation of RB. The co‐treatment with CDK4/CDK6 and AKT inhibitors demonstrated the inhibition of RB‐positive, but RB‐deficient, breast cancer cells.^36^ The binding of palbociclib with PI3K inhibitors has a synergistic/additive effect on cell growth inhibition.^37^ This combination inhibits RB hyperphosphorylation by inhibiting the cyclin D1/CDK4/CDK6 complex, inducing cell cycle arrest and inhibiting tumor growth. Few studies have compared this with palbociclib in glioma applications. For the drug alone, palbociclib quickly and effectively inhibits the proliferation without affecting the cell viability of glioma stem cell lines.^38^ The combination therapy of CDK4/CDK6 and mTOR inhibitors resulted in the synergistic growth arrest of diffuse bridge glioma cells.^14^ This is a classic combination treatment with MOTR inhibitors, which has various combinations in a variety of tumors. However, we found that almost no attempt has been performed for other treatment combinations. Previous experiments have found that AQB is also an inhibitor with a prospect of combination therapy, and that its G1/S phase cycle inhibition effect is more suitable for use with palbociclib. In order to determine whether AQB can actually enhance the inhibition of the cell cycle by palbociclib, in the subsequent experiments, we elucidated the molecular mechanism of the combination of AQB and palbociclib inhibiting the growth of glioma models from the following points. The combination of AQB and palbociclib inhibited the cell proliferation and colony formation, the migration of glioma cells in vitro, the progression from G1 to S phase in tumor cells, and the formation of intracranial tumors. In the present study, it was observed that there was a significant reduction in colony formation, and a more pronounced inhibition of the cell cycle in the combination treatment group, when compared with the control group. For the cell cycle indicators, we found that the combination treatment group inhibited the expression of CDK4/CDK6 and inhibited the phosphorylation of phosphorylated retinoblastoma (p‐RB), thereby inhibiting the G1‐to‐S phase transition. Finally, the effect of inhibiting the proliferation of tumor cells was achieved. However, it was also found that the ability of palbociclib to penetrate the blood‐brain barrier (BBB) is not good.^39^ Hence, this is also a question worth discussing, because the ability of AQB drugs to penetrate the BBB has not been carefully studied. Therefore, we intend to research this aspect in future experiments.

Wnts are particularly interesting regulators, because a key component of their signaling pathway, beta‐catenin, also functions as a component of the cadherin complex, which controls cell‐cell adhesion and influences cell migration.^40^ In a previous study, we found that AQB upregulates APC2, which is a target gene of HOTAIR‐PRC2, leading to the degradation of β‐catenin, and resulting in the inhibition of Wnt/β‐catenin signaling.^9^ We also learned that when CDK6 expression is high, the tumor is often accompanied by the activation of the Wnt /β‐catenin pathway, and palbociclib reduces the Ser9‐GSK3β phosphorylation and ultimately induces β‐catenin degradation.^20^ Therefore, it was considered that Wnt/β‐catenin signal transduction would be more significantly inhibited after the combined application. As mentioned above, there was a decrease in β‐catenin and p‐β‐catenin protein, and also a decrease in migration ability. In summary, these present data suggest that the combination of AQB and palbociclib inhibits cell migration.

## 4 CONCLUSION

The antitumor combination of HOTAIR‐EZH2 inhibitor AQB and CDK4/6 inhibitor palbociclib is more effective than either drug alone. The preset data suggest that AQB and palbociclib synergistically blocks the G1 phase of U87 and N33 cells, thereby inhibiting the critical point of cell proliferation, and reducing the migration and invasion of glioma cells by inhibiting Wnt/β‐catenin signaling. This also shows that the combination of AQB and palbociclib would have a very effective anticancer effect, since AQB is easy to synthesize and easy to mass produce, and palbociclib is presently being used in some clinical trials of tumors. Therefore, this is expected to provide a new method for treating cancer.

### 4.1 Methods

#### 4.1.1 Human tumor samples

The tumor samples used in the study were obtained from patients who were operated in The Second Affiliated Hospital of Nanchang University, Jiangxi. The NBTs used as control are composed of brain tissue obtained during the surgery of intractable epilepsy cases. The use of human gliomas and normal tissues were approved by the medical ethics committee of the Second Affiliated Hospital of Nanchang University and were performed in accordance with the approved guidelines. Informed consents were obtained from the patients. The tissues both tumor and control were snap‐frozen in liquid nitrogen and stored at −80°C.

#### 4.1.2 Cell culture and drugs

The human glioma cell line U87 was purchased from the American Type Culture Collection (ATCC, Manassas, VA), and the primary patient‐derived GBM cells N33 were provided by Professor Fan (Beijing Key Laboratory of Gene Resource and Molecular Development, Laboratory of Neuroscience and Brain Development, Beijing Normal University) (long noncoding RNA NEAT1, regulated by the EGFR pathway, contributes to GBM progression through the Wnt/β‐catenin pathway by scaffolding EZH2). The cell lines used in the experiment were cultured in Dulbecco's modified Eagle medium (DMEM; Gibco,Carlsbad, CA), containing 10% fetal bovine serum (Gibco,Carlsbad, CA), and grown at 37°C with 5% CO_2_. AQB was synthesized by Wuxi AppTec, and palbociclib was purchased from Shanghai Selleck.

#### 4.1.3 Protein mass spectrometry

Five dishes of DMSO‐ and AQB‐treated N33 cells were added to the cell lysate, followed by immunoprecipitation with HA‐Sepharose. Then, the immunoprecipitates were eluted and separated on a 6% polyacrylamide sodium dodecyl‐sulfate polyacrylamide gel electrophoresis (SDS‐PAGE) gel. Afterward, the gel was incubated with Coomassie Brilliant Blue and destained. Next, the protein bands were excised from the Coomassie‐stained gels and destained, and subjected to the mass spectrometric analysis (LC‐MS/MS). The process raw map files were obtained using the Proteome Discoverer 2.1 (Thermo Scientific, San Jose, CA) software, and finally, credible qualitative results were obtained.

#### 4.1.4 Plasmid and siRNA transfection

CWF19L1 and a control plasmid (obtained from Genechem Shanghai, China) were obtained from siRNA‐CWF19L1 (available from GenePharma, Shanghai, China; where siRNA is small interfering RNA) for the in vitro experiments. A total of 200 000 cells were seeded to 6‐well plates, and the siRNA transfection and plasmid transfection were performed using a Lipofectamine® 3000 Transfection Kit (Invitrogen,Carlsbad, CA), according to manufacturer's instructions. The final siRNA concentration was 10 nmol/L.

#### 4.1.5 Immunoprecipitation (ChIP) and ChIP‐qPCR analysis

The ChIP assays were performed using a ChIP assay kit (Beyotime,Shanghai, China)). The set Chip qPCR primers were as follows: (1) Forward: 5′‐TTCAGTCTTGAGTGCTACTCCTGG‐3′ and Reverse: 5′‐GTTCTGGCTTTTCCC‐ACAGCCTA‐3′; (2) Forward: 5′‐TATTTAGATGCCCTTGATGAGTCT‐3′ and Reverse: 5′‐GCCCTCCTACCTATTTGGTTTTA‐3′; (3) Forward: 5′‐ATCTGAATCAAAACAAGCCCCAA‐3′ and Reverse: 5′‐GTGCGGACAACAGGAATACATCTC‐3′; (4) Forward: 5′‐AACCCGACATTGT‐GCTCTGTA‐3′ and Reverse: 5′‐TACTTACGGAGACCGCCGA‐3′.

#### 4.1.6 RNA sequencing and microarray data samples

The gene expression data sets and related clinical data can be downloaded from the following websites: TCGA (https://xenabrowser.net/hub/) and CGGA (http://www.cgga.org.cn).

#### 4.1.7 Cell viability, colony formation, and Transwell assay

CCK‐8 (Dojindo, Japan) was used to assess the cell viability. On the first day, 2000 cells per well were placed in a 96‐well plate. After 48 hours of drug treatment, CCK‐8 was added for 2 hours at 37°C with 5% CO_2_ and detected using a microplate reader. The colony formation assay was initially inoculated with 300 cells per well in a 6‐well plate. After 14 days of drug treatment, the colonies were fixed with 4% paraformaldehyde, followed by crystal violet staining. The Transwell assays were performed using a Transwell membrane without Matrigel. A total of 100 000 cells in 100 mL of serum‐free DMEM were added to the upper compartment of the chamber and treated with drugs. The lower compartment was filled with 0.5 ml of FPS per well. After incubation at 37°C for 24 hours with 5% CO_2_, cells in the upper chamber were carefully removed using a cotton swab, fixed with 4% paraformaldehyde, and stained with crystal violet. The experiment was repeated for three times, and was counted by optical microscopy (100×) each time. The filter migrated five different fields of view of cells.

### 4.2 Flow cytometry

The effect of drug treatment on the cell cycle distribution of U87 and N33 was assessed by flow cytometry using propidium iodide staining. The treatment with DMSO, AQB and palbociclib alone, or AQB and palbociclib for 48 hours was performed. Then, cells were washed with PBS, digested with trypsin, collected and washed twice with IX PBS, and mixed with 1 ml of cell tissue fixative and fixed. Afterward, cells were treated with 1× propidium iodide staining buffer (containing 50 µg/mL of propidium iodide, 10 µg/mL of RNAse and 0.05% NP40). The samples were collected using a BD FACSCalibur, and analyzed for cell cycle distribution using the FlowJo and Modfit software.

#### 4.2.1 Western blot analysis and real‐time PCR

In the large dish of U87 and N33 cells over 70%, the drug was added and fixed for 2 days. Then, the protein was extracted, electrophoresed using a 10% SDS‐polyacrylamide gel, and transferred onto a polyvinylidene difluoride (PVDF) membrane. Afterwards, the PVDF membrane was blocked for 1 hour and incubated with a primary antibody at 4°C overnight. The primary antibodies used were as follows: anti‐CWF19L1 (diluted at 1:1000; Invitrogen，Carlsbad, CA); anti‐CDK4, anti‐CDK6, anti‐p‐RB (diluted at 1:1000; Abclonal，Boston，MA), anti‐RB (diluted at 1:1000, SAB,MD), anti‐CDK1, CDK2, cyclin D1, and cyclin E1 (diluted at 1:1000; Proteintech， Chicago,IL). The protein bands were detected using a SuperSignal Protein Assay Kit (Pierce，IL). The band densities of specific proteins were quantified after normalization to the density of the GAPDH band. The total RNA was extracted from these cells using a TRIzol® reagent (Invitrogen, Carlsbad, CA), according to manufacturer's instructions. The total RNA (2 µg) was reverse transcribed into cDNA using a template for reverse transcription. The cDNA was directed against each replicate internal control gene (GAPDH). The primers used were as follows: CWF19L1: Forward: 5'‐CCCAAGTGTGTGGGGAACTTT‐3' and Reverse: 5'‐GGTTTCGATATGGAAGCCTCTC‐3; CDK4: Forward:5'‐ATGGCTACCTCGATATGAGC‐3' and Reverse: 5'‐CATTGGGGACTCTCACACTCT‐3; CDK6: Forward: 5'‐TCTTCATTCACACCGAGTAGTGC‐3' and Reverse: 5'‐TGAGGTTAGAGCCATCTGGAAA‐3'.

#### 4.2.2 Nude mouse intracranial model

For the GBM PDX models, the tumors were removed from patients with GBM. The tumor tissue was cut into small sections and subcutaneously implanted into female BALB/c‐nu mice. After 4 weeks, the PDX model was successfully established, and a 5‐week‐old female nude mouse (Institute of Cancer Research, Chinese Academy of Medical Sciences) was injected with a total of the previously obtained PDX primary cells of each mouse under the guidance of a stereotactic device. After 1 week, 1 × 10^5^ cells were obtained from the DMSO group, AQB group (100 mg/kg), and palbociclib group (100 mg/kg). Palbociclib and AQB combination (palbociclib 100 mg/kg and AQB 100 mg/kg; *n* = 6 per group) was administered once every 2 days, followed by bioluminescence imaging, in order to detect the intracranial tumor growth at 7, 14, and 21 days. In the survival study, these tumor‐bearing mice were monitored daily until the animals were on the verge of death and euthanized.

#### 4.2.3 H&E staining, immunohistochemistry, and confocal imaging

Paraffin‐embedded tissue sections were used for the H&E staining and immunohistochemistry analysis. After dewaxing and rehydration, antigen retrieval was performed using sodium citrate buffer, and the sections were incubated with the primary antibody (1:200 dilution) overnight at 4°C. The biotinylated secondary antibody was incubated for 1 hour at 37°C. The horseradish peroxidase–labeled monoclonal antibody was incubated at 37°C for 40 minutes, developed, counterstained with hematoxylin, and visualized by light microscopy. The confocal imaged cells were fixed with paraformaldehyde using a phosphate buffer of 1% bovine serum albumin blocked with anti‐CWF19L1 (1:100 dilution; Invitrogen,Carlsbad, CA), F‐actin (1:100 dilution; Life Technologies,Carlsbad, CA), and anti‐CDK4 and anti‐CDK6 (1:200 dilution; Abclonal，Boston，MA). The primary antibody marker was detected by Alexa Fluor 488 or 594 conjugated secondary antibody (Alexa Fluor, Proteintech， Chicago,IL), and confocal imaging was performed on an Olympus FluoView 1200 system.

### 4.3 Statistical analysis

The experimental data were statistically significant, as determined by two‐tailed Student's *t*‐test or ANOVA for functional analysis. The Kaplan‐Meier survival curve was plotted to determine the survival curve. The statistical analysis of the data was performed using the SPSS 20.0 software and GraphPad Prism 8. All experimental data were presented as the mean ± standard deviation (SD) of three independent experiments. *P* < .05 was considered statistically significant.

## AUTHOR CONTRIBUTIONS

Jin Shi performed experiments. Miaojing Wu and Shigang Lv wrote the manuscript. Yang Den, Hongyu Zhao, Kuanxun Li, and Xianggan Wang analyzed the data. Minhua Ye designed and supervised the study. All authors read and approved the final manuscript. Yansheng Li provided many good comments on article modification.

## FUNDING INFORMATION

Natural Science Foundation of Jiangxi Province; Grant Number: 20171ACB20035; National Natural Science Foundation; Grant Numbers: 81760445, 81660420, 81960456, and 81760446.

## CONFLICT OF INTEREST

The authors declare that there is no conflict of interest that could be perceived as prejudicing the impartiality of the research reported.

## Supporting information

Supplement Information

Supplement Information

Supplement Information

## Data Availability

The analyzed data sets generated during the study are available from the corresponding author on reasonable request.
