# Supplementary figures and images for: HOTAIR‐EZH2 inhibitor AC1Q3QWB upregulates CWF19L1 and enhances cell cycle inhibition of CDK4/6 inhibitor palbociclib in glioma
Source: Clin Transl Med. 2020 Apr 29;10(1):182–98. doi: 10.1002/ctm2.21 (PMC7240863; doi:10.1002/ctm2.21)

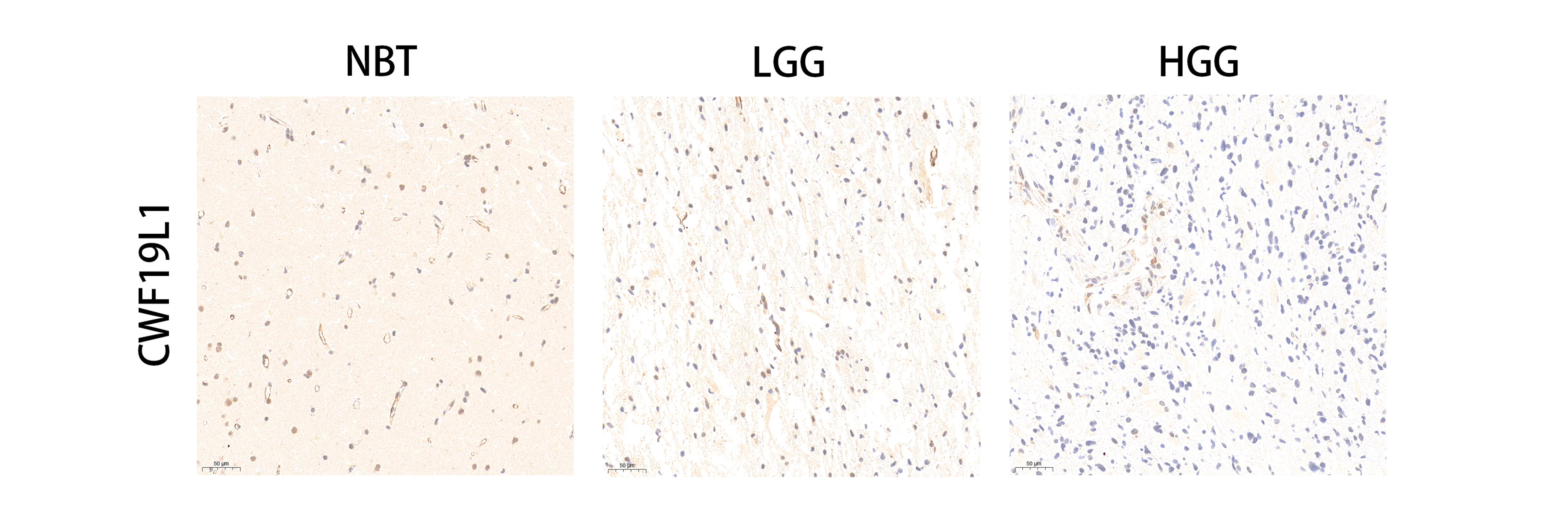

Supplement: Supplementary file 1 — Supplement Information [file CTM2-10-182-s001.tif]

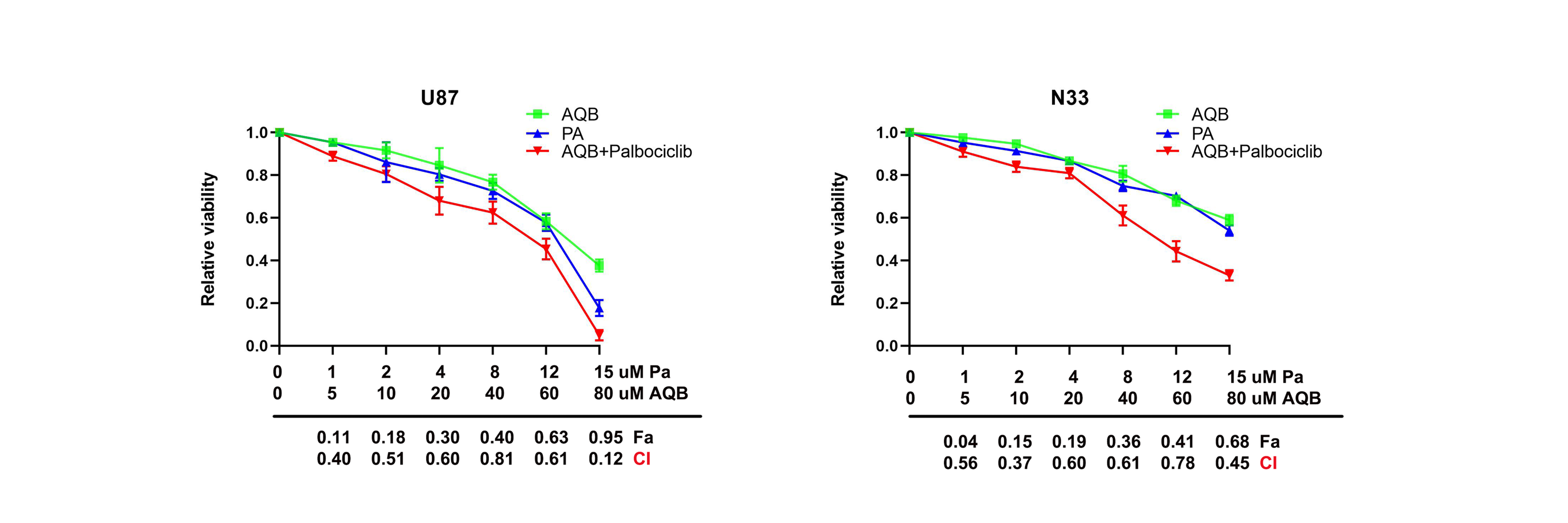

Supplement: Supplementary file 2 — Supplement Information [file CTM2-10-182-s002.tif]

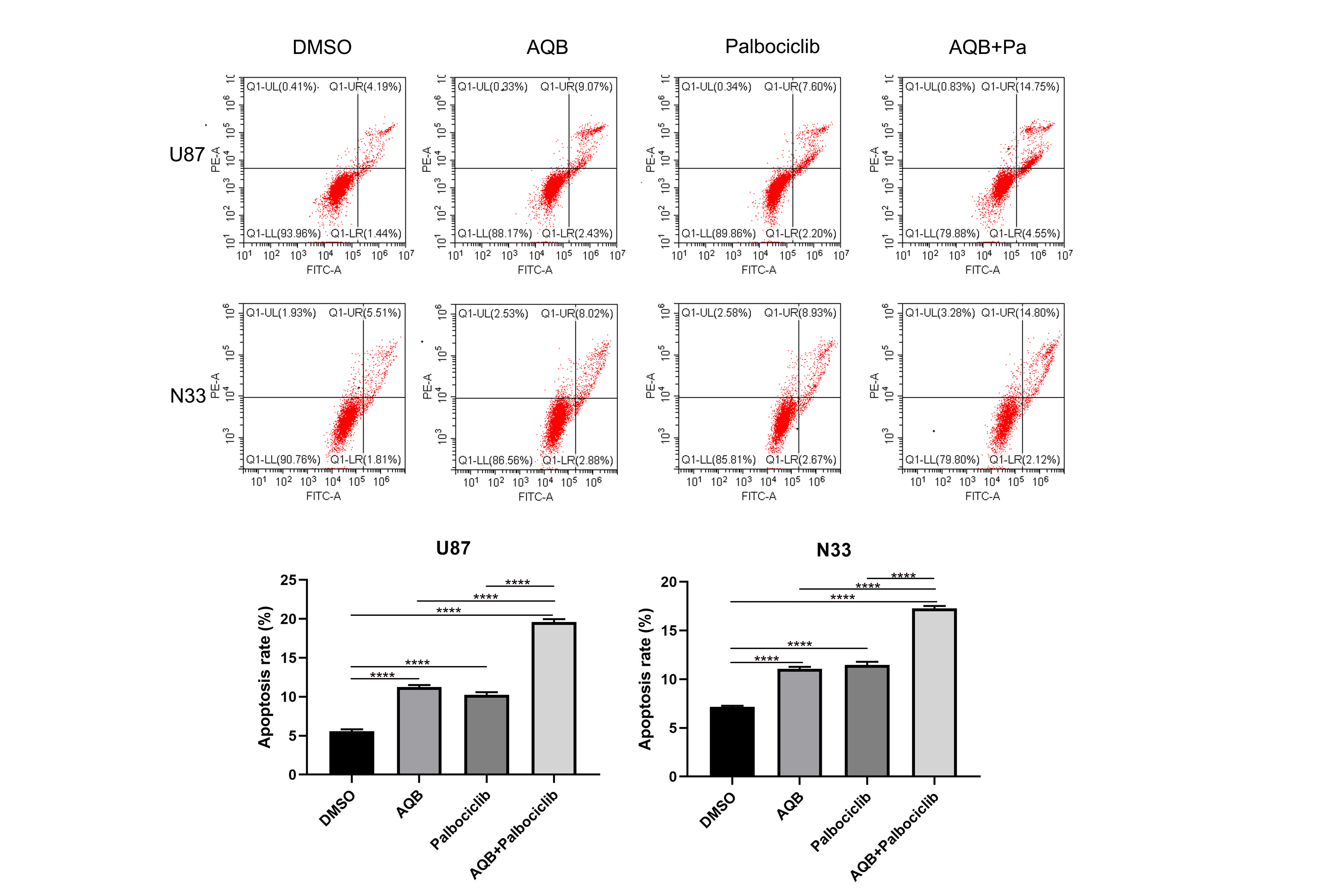

Supplement: Supplementary file 3 — Supplement Information [file CTM2-10-182-s003.tif]
